# Synthetic DNA and RNA Programming

**DOI:** 10.3390/genes10070523

**Published:** 2019-07-11

**Authors:** Patrick O’Donoghue, Ilka U. Heinemann

**Affiliations:** 1 Department of Biochemistry, The University of Western Ontario, London, ON N6A 5C1, Canada; 2 Department of Chemistry, The University of Western Ontario, London, ON N6A 5C1, Canada

**Keywords:** genetic code expansion, genome synthesis, genome editing, microRNA, protein modification, RNA metabolism, tRNA, synthetic biology, unnatural amino acids, unnatural nucleotides

## Abstract

Synthetic biology is a broad and emerging discipline that capitalizes on recent advances in molecular biology, genetics, protein and RNA engineering as well as omics technologies. Together these technologies have transformed our ability to reveal the biology of the cell and the molecular basis of disease. This Special Issue on “Synthetic RNA and DNA Programming” features original research articles and reviews, highlighting novel aspects of basic molecular biology and the molecular mechanisms of disease that were uncovered by the application and development of novel synthetic biology-driven approaches.

Synthetic biology is a broad and emerging discipline that capitalizes on recent advances in molecular biology, genetics, protein and RNA engineering, as well as omics technologies. Together these biotechnologies have transformed our ability to reveal the biology of the cell and the molecular basis of disease. This special issue of *Genes* on “Synthetic DNA and RNA Programming” features 12 original research articles and reviews that highlight novel aspects of basic molecular and cellular biology, uncovered by the application and development of synthetic biology-driven approaches.

The approaches highlighted here involve programming genes, RNAs, and proteins, both in the test tube and in living cells, to explore areas of molecular biology related to genetic code evolution [1,2] and genetic code expansion [3,4,5], including applications in programming protein modifications [6,7], RNA metabolism [8,9], and genetic systems for genome engineering [10,11] and biocontainment of modified microorganisms [12]. These contributions showcase both the diversity of research approaches emerging as synthetic biology tools and the extreme level of detail with which these tools enable studies and the manipulation of individual protein modifications or cellular pathways. The contributed articles cluster into four thematic categories: *genetic code expansion*, *genetic code evolution*, *novel genetic systems and molecular tools*, and *RNA programming*.

## 1. Expanding the Genetic Code 

Genetic code expansion studies focus on methods that enable the production of proteins with amino acids beyond the canonical or standard 20 genetically encoded amino acids. Cells with engineered and expanded genetic codes can produce proteins with 21 [13] or even 22 [14,15] different genetically encoded amino acids. These additional amino acids allow the introduction of new and specific chemically functionalized side chains.

Genetic code expansion normally requires the addition of a new aminoacyl-tRNA synthetase and tRNA pair. Efficient and high-fidelity genetic encoding requires that the AARS tRNA pair is mutually orthogonal to the endogenous AARSs and tRNAs in the cell. These methods normally reassign the meaning of a stop codon, usually UAG, to the direct incorporation of an additional, non-canonical amino acid (ncAA). The most commonly used orthogonal pairs include the archaeal enzymes tyrosyl-tRNA syntetase (TyrRS) [13], the pyrrolysyl-tRNA synthetase (PylRS) [16,17], and the phosphoseryl-tRNA synthetase (SepRS) [18]. Balasuriya et al. used the phosphoserine system to produce highly active human kinases with programmed phosphorylation from facile *Escherichia coli* expression systems [7]. The authors used these reagents to identify a specific phosphorylation site on protein kinase B (Akt1) that interferes with a clinically relevant kinase inhibitor.

Genetic code expansion also enables the incorporation of other posttranslational modifications at specific or programmed sites in proteins. Umehara et al. [6] used a mutant of the PylRS tRNA^Pyl^ orthogonal pair to genetically encode *N^ε^*-acetylated lysine into the *E. coli* alanyl-tRNA synthetase. The resulting acetylated AlaRS was catalytically deficient. The authors next used in vivo assays to determine that two Cob deacetylases were able to remove the K73 acetylation, identifying a novel regulatory pathway to control AlaRS activity.

The study from Hoffmann et al. focused on the central role of tRNAs in genetic code expansion [3]. Specifically, this review article addressed two ‘fundamentally different translation systems’ that evolved in natural organisms. These include the systems that genetically encode selenocysteine and pyrrolysine, the so-called 21st and 22nd amino acids. Design and engineering of tRNA variants for optimal genetic code expansion were examined, as were synthetic biology applications of genetic code expansion.

Finally, Gang et al. focused on the application of peptides with ncAAs as antibiotics. Antibiotic development is a matter of urgent clinical need and one of the most promising areas is related to the development of synthetic cyclic peptides. These compounds are often inspired by natural products, but through the use of genetic code expansion, ncAAs can be included in these antibiotic peptides [4]. The review article from Gang et al. focused on methods for peptide cyclization and applications of cyclic peptides.

## 2. Genetic Code Evolution

Although Crick’s frozen accident hypothesis once envisioned a perfectly interpreted genetic code [19], recent studies have found that cells tolerate surprisingly high levels of amino acid misincorporation [20]. The next pair of featured articles [1,2] investigated biochemical mechanisms that regulate translation fidelity.

Berg et al. [1] provided the first detailed characterization of human tRNA^Ser^ identity elements. These are the critical nucleotides that are essential for tRNA recognition by the cognate aminoacyl-tRNA synthetase and determine the serine accepting identity of this tRNA. The tRNA identity elements define the correspondence of amino acids with anticodons and thus are fundamental to the accurate decoding of the genetic code. Using a unique in vivo assay, relying on the misincorporation of serine at proline codons in yeast, this work identified the evolution of new identity elements in the human tRNA^Ser^.

Similarly, the article by Rathnayake et al. [2] also highlighted the fact that the mechanisms and molecular entities tasked with ensuring the faithful interpretation of genetic code have evolved over time into idiosyncratic variants. The aspartyl-tRNA synthetase (AspRS) enzymes occur as two variants, the discriminating AspRS, which exclusively charges Asp to tRNA^Asp^, as well as a non-discriminating variant (ND-AspRS). ND-AspRS charges Asp onto tRNA^Asn^ in a pathway generating Asn-tRNA^Asn^ via Asp-tRNA^Asn^. In some bacteria, both AspRS and ND-AspRS are capable of not only charging Asp to tRNA^Asp^ and tRNA^Asn^, but also of have an unexpected glutamyl-tRNA synthetase (GluRS)-like activity. In these cases, AspRS acts as a GluRS, and is able to ligate Glu to tRNA^Glu^ but not to tRNA^Asp^. Although the AspRS enzyme targets a non-cognate tRNA, amino acid misincorporation does not occur, thus preserving translation fidelity.

## 3. Novel Genetic Systems and Molecular Tools

Gordon et al. opened the door to studying the unusual biology of yeast of the genus *Metschnikowia* [11]. These yeasts have a genetic code variation in which the leucine CUG codons are instead decoded as serine. The authors developed a new and efficient transformation system for *Metschnikowia* and an additional 21 yeast species, providing new platforms for genome synthesis and engineering efforts.

Protein expression is an essential methodology for biochemical studies, including those mentioned above, as well as for the production of biopharmaceuticals such as antibodies and therapeutic peptides. Chen et al. [10] used a high-throughput approach to screen for the impact of the expression of any *E. coli* gene on cell growth. The most significant impact on *E. coli* growth was observed upon the overexpression of membrane proteins. Interestingly, the authors found that for certain proteins, the use of lower copy number plasmids stabilized cell growth rate and increased overall recombinant protein yields. The impact on cell growth could also often be remedied by amino acid supplementation. Using a related methodology, Schwark et al. used a fluorescent protein reporter system and investigated an *E. coli* release factor deletion strain with a highly efficient archaeal orthogonal AARS tRNA pair. Their data suggested that the engineered strain will be a highly useful tool for applications requiring the incorporation of multiple ncAAs [5].

A major ethical responsibility for synthetic biologists is the ability to retain control or confinement of genetically modified microorganisms. This drove the field to develop fascinating and effective methods for containing microorganisms in the lab that are commonly referred to as biocontainment. Some of these approaches included the use of genetic code expansion to create synthetic auxotrophs, such as an *E. coli* strain dependent on ncAAs for growth [21]. Diwo et al. further extended this idea with their novel formulation of an ‘alien genetic code’ [12]. The authors presented the concept that an ideal biocontainment system would involve the creation of microbes with genetic codes that are totally incompatible or ‘alien’ to the natural genetic code. The construction of such an organism is considered to be achieved via the directed evolution of an existing cell or through de novo construction of ‘synthetic’ genomes and cells.

## 4. RNA Programming

RNAs play important roles in controlling translation, yet there are still limited tools to study and engineer RNAs and their activity in cells. Turk et al. developed a novel reporter system to quantify the activity of microRNAs (miRNAs) in living cells [9]. While microRNAs can be quantified by methods such as quantitative PCR, a variable and unknown portion of cellular miRNAs is inactive. To assess the amount of active miRNA in the cell, the authors developed a GFP-based reporter system that allows for time- and space-resolved monitoring of active miRNAs in single cells.

Another limitation of analyzing RNAs is the limitation of commercially available polymerases to extend RNAs in the forward 5′ to 3′ direction. Chen et al. [8] reviewed recent progress made towards the characterization and engineering of tRNA^His^ guanylyltransferase homologs capable of reverse 3′ to 5′ nucleotide polymerization. Tools and enzymes with broadened RNA substrates and extended template-dependent reverse polymerization activity were discussed and will be invaluable for RNA labeling, engineering, and analysis efforts.

In summary, this collection of articles represents new directions in multiple areas of interest to synthetic biologists. From expanding the number of genetically encoded amino acids to creating new genetic tools in diverse microbes, these articles are each exemplars of the sophisticated synthetic biology approaches that produce cells with new capabilities, enabling the production of designer proteins and RNAs. Some of these tools reveal molecular events in living cells that were previously inaccessible.
